# Diagnostic Performance of [^18^F]TFB PET/CT Compared with Therapeutic Activity [^131^I]Iodine SPECT/CT and [^18^F]FDG PET/CT in Recurrent Differentiated Thyroid Carcinoma

**DOI:** 10.2967/jnumed.123.266513

**Published:** 2024-02

**Authors:** David Ventura, Matthias Dittmann, Florian Büther, Michael Schäfers, Kambiz Rahbar, Daniel Hescheler, Michael Claesener, Philipp Schindler, Burkhard Riemann, Robert Seifert, Wolfgang Roll

**Affiliations:** 1 Department of Nuclear Medicine, University Hospital Münster, Münster, Germany;; 2 West German Cancer Centre, Münster, Germany;; 3 Department of Nuclear Medicine, St. Marien Hospital Lünen, Lünen, Germany;; 4 European Institute for Molecular Imaging, University of Münster, Münster, Germany;; 5 Clinic for Radiology, University and University Hospital Münster, Münster, Germany;; 6 Department of Nuclear Medicine, University Hospital Essen, Essen, Germany; and; 7 West German Cancer Centre, Essen, Germany

**Keywords:** [^18^F]TFB, radioactive iodine therapy, DTC, [^18^F]FDG, PET

## Abstract

[^18^F]tetrafluoroborate ([^18^F]TFB) is an emerging PET tracer with excellent properties for human sodium iodide symporter (NIS)–based imaging in patients with differentiated thyroid cancer (DTC). The aim of this study was to compare [^18^F]TFB PET with high-activity posttherapeutic [^131^I]iodine whole-body scintigraphy and SPECT/CT in recurrent DTC and with [^18^F]FDG PET/CT in suspected dedifferentiation. **Methods:** Twenty-six patients treated with high-activity radioactive [^131^I]iodine therapy (range, 5.00–10.23 GBq) between May 2020 and November 2022 were retrospectively included. Thyroid-stimulating hormone was stimulated by 2 injections of recombinant thyroid-stimulating hormone (0.9 mg) 48 and 24 h before therapy. Before treatment, all patients underwent [^18^F]TFB PET/CT 40 min after injection of a median of 321 MBq of [^18^F]TFB. To study tracer kinetics in DTC lesions, 23 patients received an additional scan at 90 min. [^131^I]iodine therapeutic whole-body scintigraphy and SPECT/CT were performed at a median of 3.8 d after treatment. Twenty-five patients underwent additional [^18^F]FDG PET. Two experienced nuclear medicine physicians evaluated all imaging modalities in consensus. **Results:** A total of 62 suspected lesions were identified; of these, 30 lesions were [^131^I]iodine positive, 32 lesions were [^18^F]TFB positive, and 52 were [^18^F]FDG positive. Three of the 30 [^131^I]iodine-positive lesions were retrospectively rated as false-positive iodide uptake. Tumor-to-background ratio measurements at the 40- and 90-min time points were closely correlated (e.g., for the tumor-to-background ratio for muscle, the Pearson correlation coefficient was 0.91; *P* < 0.001; *n* = 49). We found a significant negative correlation between [^18^F]TFB uptake and [^18^F]FDG uptake as a potential marker for dedifferentiation (Pearson correlation coefficient, −0.26; *P* = 0.041; *n* = 62). **Conclusion:** Pretherapeutic [^18^F]TFB PET/CT may help to predict the positivity of recurrent DTC lesions on [^131^I]iodine scans. Therefore, it may help in the selection of patients for [^131^I]iodine therapy. Future prospective trials for iodine therapy guidance are warranted. Lesion [^18^F]TFB uptake seems to be inversely correlated with [^18^F]FDG uptake and therefore might serve as a dedifferentiation marker in DTC.

## Visual Abstract

**Figure:**
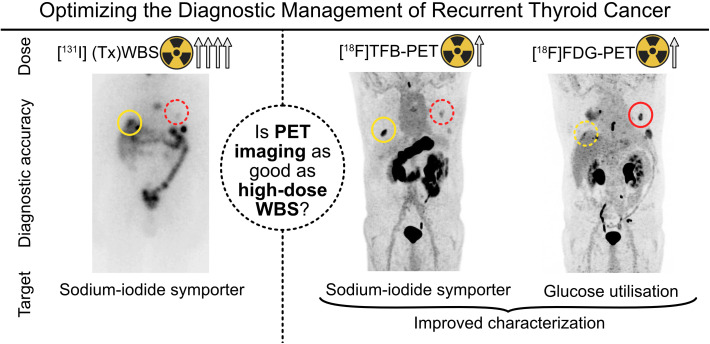

The incidence of differentiated thyroid carcinoma (DTC) is currently increasing at a rate of approximately 3% per year, with papillary thyroid carcinoma being the major histologic tumor entity, followed by follicular thyroid carcinoma (1*,*^2^). After standard treatment with thyroidectomy and radioactive [^131^I]iodine therapy (RAI), approximately 90% of DTC patients have a normal life expectancy. However, up to 50% will develop lymph node metastases and about 10% will develop distant metastases (3). Importantly, dedifferentiation may occur in the course of the disease, accompanied by a decrease or loss of expression of the sodium iodide symporter (NIS) and the subsequent failure of [^131^I]iodine-based theranostics (4). These radioiodine-refractory DTC (RRDTC) patients have poorer survival rates (2). Therefore, a multimodal approach including various imaging modalities is recommended in patients with tumor recurrence or suspected RRDTC to guide treatment decisions (5).

NIS-based imaging is a standard procedure for the detection of suspected DTC recurrence with the therapeutic/diagnostic application of [^131^I]iodine for whole-body scintigraphy (WBS) and SPECT/CT (WBS-SPECT/CT) (6). However, the low signal-to-noise ratio and the low spatial resolution of WBS-SPECT/CT in combination with [^131^I]iodine limit their sensitivity. This issue may cause the “thyroglobulin elevated and negative scintigraphy” (TENIS) syndrome (7). In addition, [^131^I]iodine as a β-emitter involves comparably high radiation exposure (8). As yet, positron-emitting [^124^I]iodine is only available in a few centers and, again, is associated with a low signal-to-noise ratio (9). A promising alternative PET radiotracer for targeting NIS is [^18^F]tetrafluoroborate ([^18^F]TFB), which has a high signal-to-noise ratio and involves lower radiation exposure (10). It was shown previously that [^18^F]TFB PET/CT offers equivalent diagnostic accuracy as [^124^I]iodine PET/CT (11). We recently demonstrated that [^18^F]TFB PET/CT has high accuracy compared with [^131^I]iodine-based diagnostic SPECT/CT (12). However, there is a lack of knowledge regarding the clinically relevant comparison of [^18^F]TFB PET/CT with posttherapeutic SPECT/CT in recurrent DTC (13).

Differentiated metastases of thyroid cancer still express NIS and thereby show iodide uptake with low glucose metabolism, whereas dedifferentiated metastases have lost NIS expression and do not trap iodide sufficiently but have a high glucose consumption (14*,*^15^). [^18^F]FDG PET/CT uptake correlates with the degree of DTC dedifferentiation and therefore has already established its diagnosis (16). The performance of [^18^F]TFB PET/CT in dedifferentiating DTC is unknown. In addition, there is a lack of evidence regarding the optimal imaging time point after tracer injection for [^18^F]TFB PET/CT (10).

Therefore, the aim of this retrospective study was to evaluate the accuracy and optimal time point of [^18^F]TFB PET/CT in patients with suspected recurrence of DTC in comparison to clinically approved therapeutic WBS-SPECT/CT (TxWBS-SPECT/CT). In addition, [^18^F]TFB PET/CT is also compared with [^18^F]FDG PET/CT to assess dedifferentiation in DTC.

## MATERIALS AND METHODS

### Patients

This single-center retrospective study included 26 patients with recurrent DTC treated with high-activity RAI (range, 5.00–10.23 GBq) between May 2020 and November 2022. Before RAI, all included patients underwent [^18^F]TFB PET/CT, with its previously demonstrated higher diagnostic accuracy, in routine clinical practice to evaluate potential localized treatment options. High-activity therapeutic RAI was recommended by the local tumor board on the basis of international guidelines (5). All patients underwent TxWBS-SPECT/CT after RAI. Additional [^18^F]FDG PET/CT was performed if dedifferentiation was suspected. Detailed characteristics of the patients are summarized in Table 1.

**TABLE 1.** Patient Characteristics

Characteristic	*n*	%	Median	Range	95% CI
Patients*	26				
Sex					
Women	12	46.2			
Men	14	53.8			
Histology					
PTC	11	42.3			
FTC	13	50			
OTC	2	7.7			
UICC TNM (33)					
pT1	4	15.4			
pT2	7	27			
pT3	9	34.6			
pT4	3	11.5			
pTx	3	11.5			
pN0	5	19.2			
pN1	7	27			
pNx	14	53.8			
cM0	5	19.2			
cM1	16	61.5			
cMx	5	19.2			
PUL	5	19.2			
OSS	7	27			
OTH	4	15.4			
Laboratory analysis					
TG^†^ (ng/mL)			10.1	0.15–10,807	
Anti-TG^†^ (IU/mL)			17.2	<15–230	
TSH (μU/mL)			0.1	0.02–2.36	
TSH after stimulation (μU/mL)			122	51.8–424.1	
TG after stimulation (ng/mL)			61.9	0.46–21,200	
8 wk after RAI					
TG^†^			6.7	0.15–10,780	
Anti-TG^†^			15.6	<15–238	
RAI and PET/CT					
GBq of [^131^I]iodine			6,023		5,032–6,497
MBq of [^18^F]TFB			321		297–342
MBq of [^18^F]FDG			326		270–317

* Median age was 64.5 y, and range was 38–87.

† If measurable.

PTC = papillary thyroid carcinoma; FTC = follicular thyroid carcinoma; OTC = oncocytic carcinoma of thyroid; PUL = pulmonary metastases; OSS = bone metastases; OTH = other-side metastases; anti-TG = antibodies against thyroglobulin.

All patients gave written and informed consent before treatment and imaging acquisition in accordance with the Declaration of Helsinki. This retrospective study was approved by the local ethic committee (2019-615-f-S; Ethik-Kommission Westfalen-Lippe).

### [^18^F]TFB PET/CT and [^131^I]Iodine TxWBS-SPECT/CT

All patients received 2 intramuscular injections of thyrotropin alfa (Thyrogen; Sanofi) (0.9 mg) 48 and 24 h before [^18^F]TFB PET/CT and RAI. Thyroid-stimulating hormone (TSH) was assessed immediately before PET and RAI. [^18^F]TFB was produced in-house as described in detail previously (12) and administered intravenously at 3 MBq/kg of body weight. Whole-body PET acquisitions from the head to the proximal femur (acquisition speed, 2 min/bed position or 1.1 mm/s) were performed 40 min (all patients) and 90 min (23/26 patients) after injection using a Siemens Biograph 128 mCT scanner (Siemens Healthcare). A low-dose CT scan was performed for attenuation correction and anatomic correlation.

A median activity of 6.02 GBq (range, 5.00–10.23 GBq) of [^131^I]iodide was administered orally (Curium Germany GmbH) immediately after PET acquisition. Planar TxWBS scans were acquired using a high-energy parallel-collimator matrix with a 2.21-mm pixel size at a speed of 10 cm/min and a photopeak at 364 keV (±10%). Also, all patients underwent SPECT/CT from the skull base to the diaphragm and, on the basis of the TxWBS scans, SPECT/CT of additional regions using a Discovery NM/CT 670 Pro System (General Electric Co.). SPECT was acquired with a high-energy collimator matrix of 128 × 128 in the step-and-shoot mode with 20 s/step and a photopeak at 364 keV (±10%).

### Tumor-to-Background (TBR) Ratios in [^18^F]TFB PET

The maximum TBR was measured with 3 reference organs to reliably compare tracer kinetics in DTC lesions. The maximum TBR was calculated as the quotient of lesion SUV_max_ and reference SUV_mean_ (17). The reference SUV_mean_ was measured using a spheric 1.0-cm-diameter volume of interest in the thoracic aorta (blood-pool TBR) or a 3.0-cm-diameter volume of interest in the left gluteus maximus muscle (muscle TBR) or in the right apical lung lobe (lung TBR). This procedure was done for both 40- and 90-min PET scans.

### [^18^F]FDG PET/CT

[^18^F]FDG PET/CT was performed if dedifferentiated disease was suspected (5). Eligible patients were imaged after at least 6 h of fasting with blood glucose of less than 6.7 mM. Images were acquired at 60 min after the intravenous administration of body weight–adapted [^18^F]FDG (3 MBq/kg) (acquisition speed, 2 min/bed position or 1.1 mm/s) using a Siemens Biograph 128 mCT scanner. Whole-body images from the head to the proximal femur were acquired and, for attenuation correction, an additional low-dose CT scan was performed.

### Image Analysis

All available image acquisitions were reviewed in consensus by 2 experienced nuclear medicine physicians. Nonphysiological focal uptake above the background uptake of [^131^I]iodine in TxWBS and correlative SPECT/CT was considered suspicious. Similarly, nonphysiological focal [^18^F]TFB uptake was defined as uptake exceeding the background uptake (e.g., muscular uptake for cervical lesions) and considered suspicious. Focal [^18^F]FDG uptake was considered suspicious when the uptake was over the liver background uptake (18). For lesion-based analysis, the SUV_max_ was measured in each lesion for [^18^F]FDG and [^18^F]TFB at both 40 and 90 min. Image analysis for PET/CT and TxWBS-SPECT/CT was performed with Syngo.via (Siemens Healthcare).

### Biochemical Analysis

Free triiodothyronine, free thyroxine, TSH, thyroglobulin (TG), and antibodies against TG were measured on the first day before the intramuscular injection of thyrotropin alfa. Verification of stimulated TSH was performed before the application of [^18^F]TFB and [^131^I]iodide. Stimulated TG was measured 3 d after a second injection with thyrotropin alfa (Elecsys assays and cobas e 801 [Roche Diagnostics]; TG, assay, and high-sensitive TG Kryptor [BRAHMS GmbH; Thermo Fisher Scientific]).

### Statistical Analysis

Clinical and demographic parameters are presented as total number, percentage, range, and 95% CI. The Pearson correlation coefficient (*r*_p_) and the φ-coefficient (*r*_φ_) were used for continuous and binary variables, respectively. Values of greater than 0.10, 0.30, and 0.50 for |*r*_p_| and |*r*_φ_| correspond to low, intermediate, and strong positive correlations and vice versa for negative correlations. The null hypothesis was rejected if the *P* value was less than 0.05 (2-sided). Statistical analysis was performed using SPSS Statistics version 26 (SPSS Inc.).

## RESULTS

### Patient-Based Analysis

A total of 26 patients who received [^18^F]TFB PET/CT, RAI, and [^131^I]iodine TxWBS-SPECT/CT were included in this retrospective study. The indication for high-activity RAI was imaging evidence of lymph node or distant metastases on [^18^F]FDG PET/CT (22/26; 84.6%), ultrasound (2/26; 7.7%), MRI (1/26; 3.9%), and [^131^I]iodine diagnostic WBS (1/26; 3.9%). Of the 22 patients with available [^18^F]FDG PET/CT, local recurrence (3/22; 13.6%), lymph node metastases (5/22; 22.7%), lung metastases (9/22; 30.9%), bone metastases (8/22; 36.3%), or metastases to other organs (2/22; 9.1%) as well as [^18^F]FDG-positive and -negative findings were identified. The ultrasound or MRI findings were suspected cervical lymph node metastases. Local recurrence was suspected in the patient with [^131^I]iodine diagnostic WBS imaging.

**FIGURE 1.**
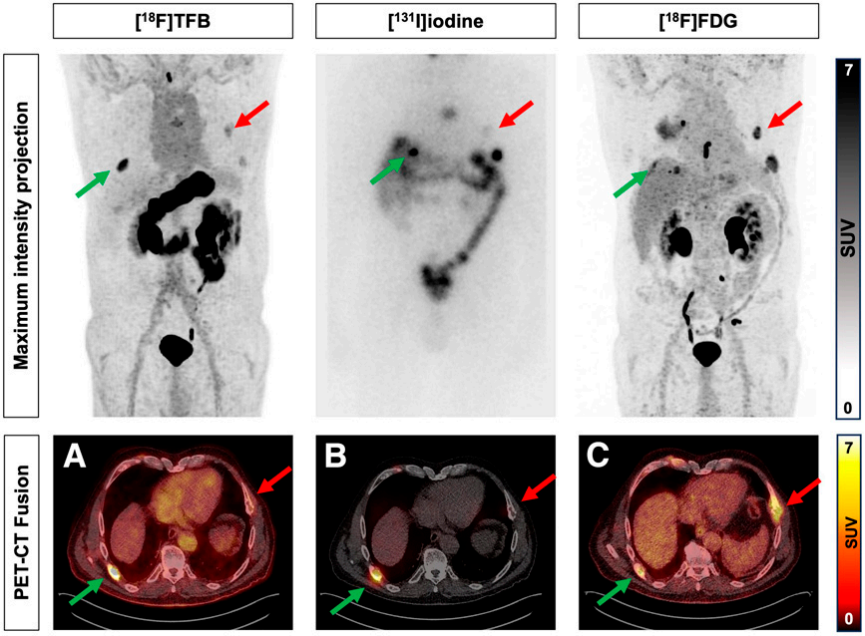
Heterogeneous disease pattern. 71-y-old man with follicular thyroid carcinoma and various bone metastases. Rib metastases show heterogeneous disease pattern: intense [^18^F]TFB (A) and [^131^I]iodine (B) uptake but low [^18^F]FDG (C) uptake (green arrow); intense [^18^F]FDG (C) uptake but no relevant [^18^F]TFB (A) and [^131^I]iodine (B) uptake (red arrows).

The median time between [^18^F]TFB PET/CT/RAI and [^131^I]iodine TxWBS-SPECT/CT was 3.8 d (95% CI, 3.3–4.2 d). A total of 25 of 26 patients (96%) underwent [^18^F]FDG PET/CT for suspicion of dedifferentiated disease. The median time between [^18^F]TFB PET/CT/RAI and [^18^F]FDG PET/CT was 32.8 d (95% CI, 18.3–47.3 d). [^18^F]TFB-, [^131^I]iodine-, and [^18^F]FDG-positive lesions were seen in 11 of 26 patients (42%), 12 of 26 patients (46%), and 11 of 25 patients (42%), respectively. On a per-patient basis, cross-tabulation with the χ^2^ test revealed a strong and significant correlation (*r*_φ_) between [^18^F]TFB and [^131^I]iodine findings (*r*_φ_ = 0.61; *P* < 0.001; *n* = 26) and a low, nonsignificant correlation between [^18^F]TFB and [^18^F]FDG findings (*r*_φ_ = 0.06; *P* = 0.902; *n* = 25). A heterogeneous disease pattern is illustrated in Figure 1.

### Lesion-Based Analysis

**FIGURE 2.**
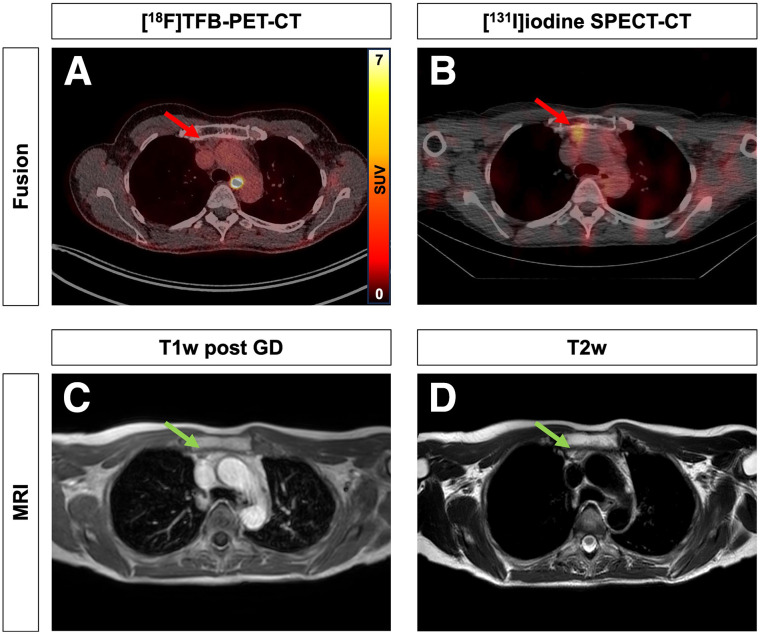
False-positive thymus uptake in [^131^I]iodine SPECT/CT. 49-y-old woman with follicular thyroid cancer. Absent [^18^F]TFB uptake (A) in [^131^I]iodine-positive (B) mediastinal mass (red arrow). Subsequent contrast-enhanced MRI showed thymus rebound (green arrow) in T1-weighted (after gadolinium) (T1w post GD) and T2-weighted (T2w) (C and D) sequences. [^131^I]iodine uptake therefore was considered to be false-positive, a conclusion that was also confirmed by further follow-up.

**FIGURE 3.**
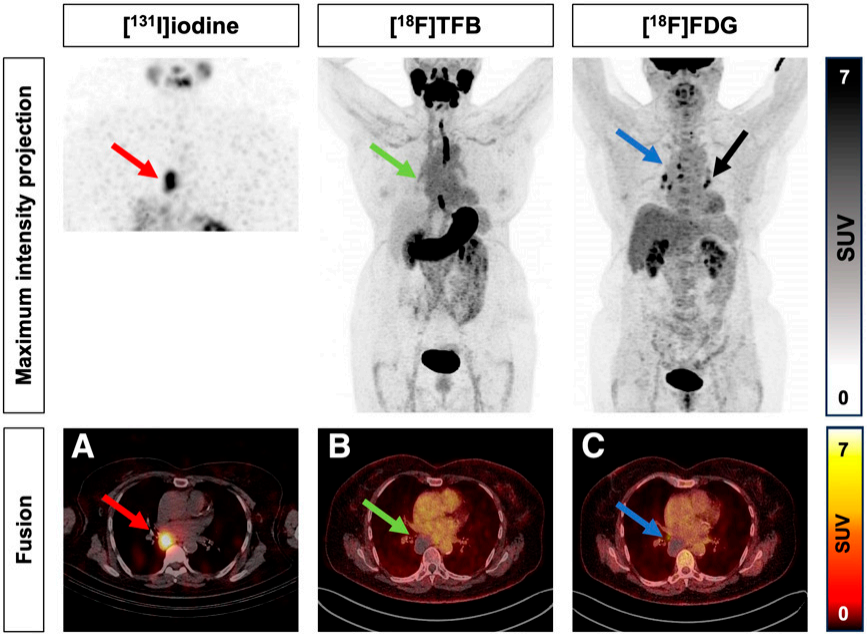
False-positive low mediastinal mass in [^131^I]iodine SPECT/CT. 67-y-old woman with papillary thyroid cancer. Intense [^131^I]iodine uptake in low mediastinal mass (red arrow, A) with absent uptake of [^18^F]TFB (green arrow, B) and [^18^F]FDG (blue arrow, C). CT scan revealed probably benign hypodense lesion. Histologic confirmation was recommended due to intense [^131^I]iodine uptake. Samples were obtained by endobronchial ultrasound-guided transbronchial needle aspiration, which revealed benign soft-tissue cells, suggesting hemorrhagic cyst. Additionally, [^18^F]FDG-positive (black arrow) and [^18^F]TFB/[^131^I]iodine–negative mediastinal lymph node metastases were visualized by maximum-intensity-projection imaging.

A total of 62 tracer-avid lesions were found in all imaging modalities; of these, 32 of 62 (52%) were [^18^F]TFB positive, 30 of 62 (48%) were [^131^I]iodine positive, and 52 of 62 (84%) were [^18^F]FDG positive. A detailed lesion-based analysis is illustrated in Supplemental Table 1 (supplemental materials are available at http://jnm.snmjournals.org). False-positive [^131^I]iodide uptake was confirmed in 3 of 26 patients (11%), as illustrated for 2 patients in Figure 2 and Figure 3.

Without the aforementioned false-positive [^131^I]iodine uptake, [^18^F]TFB PET/CT detected 5 of 62 additional lesions (8%) compared with [^131^I]iodine imaging. Detailed findings for patients, including the description of false-positive and [^131^I]iodine mismatch findings, are summarized in Table 2.

**TABLE 2.** Findings Per Patient

Patient identification	Finding	[^18^F]TFB	[^131^I]iodine TxWBS- SPECT/CT	[^18^F]FDG	Dominant tumor burden
1	Cervical and mediastinal lymph nodes	N	P	P	[^18^F]FDG
2	Lung nodules	N	N	N	N/A
3	Bone lesions and lung nodules	P	N	P	[^18^F]FDG
4	N/A	N	N	N	N/A
5	Lung nodules	N	N	P	[^18^F]FDG
6	Glottis and cervical lymph nodes	P	P	N	[^18^F]TFB/[^131^I]iodine
7	Local recurrence	P	N	P	[^18^F]TFB
8	High mediastinal lymph nodes and low mediastinal mass	N	fP*	P	[^18^F]FDG
9	Lung nodules	N	N	N	N/A
10	Local recurrence on right	P	P	N	[^18^F]TFB/[^131^I]iodine
11	Lung nodules	P	P	N	[^18^F]TFB/[^131^I]iodine
12	Lung nodules and inflammation	N	fP^†^	N	N/A
13	Lung nodules	P	P	P	[^18^F]FDG
14	Bone lesions	P	P	P	[^18^F]FDG
15	Local recurrence on right and bone lesions	P	P	N	[^18^F]TFB/[^131^I]iodine
16	N/A	N	N	N	N/A
17	Lung nodules and bone lesions	N	P	P	[^18^F]FDG
18	Bone lesions	P	P	N	[^18^F]TFB/[^131^I]iodine
19	Bone lesions	P	P	N	[^18^F]TFB
20	Lung lesions and soft-tissue lesion	N	P	P	[^131^I]iodine
21	N/A	N	N	N	N/A
22	N/A	N	N	N	N/A
23	N/A	N	N	N/A	N/A
24	Thymus and cervical lymph nodes	N	fP^‡^	P	[^18^F]FDG
25	Bone lesions	P	P	P	[^18^F]TFB/[^131^I]iodine
26	N/A	N	N	N	N/A

* Histologic verification: no malignancy was found in [^131^I]iodine-positive low mediastinal mass.

† Primary pulmonary inflammation due to chronic obstructive pulmonary disease.

‡ Follow-up: [^131^I]iodine accumulation in projection onto thymus, verification by MRI.

N = negative; P = positive; N/A = not available; fP = false-positive.

[^131^I]iodine-positive mismatch detailed patient analysis: patient 1—predominantly [^18^F]FDG-positive (SUV_max_, 8.3) tumor burden with low [^131^I]iodine uptake in solitary mediastinal lymph node metastases; patient 17—predominantly [^18^F]FDG-positive (SUV_max_, 15.8) tumor burden with low [^131^I]iodine uptake in solitary lung and bone metastases; patient 20—predominantly [^18^F]FDG-positive (SUV_max_, 5.2) tumor burden with intermediate [^131^I]iodine uptake in solitary soft-tissue metastasis.

### Quantitative Analysis of Lesion Differentiation

**FIGURE 4.**
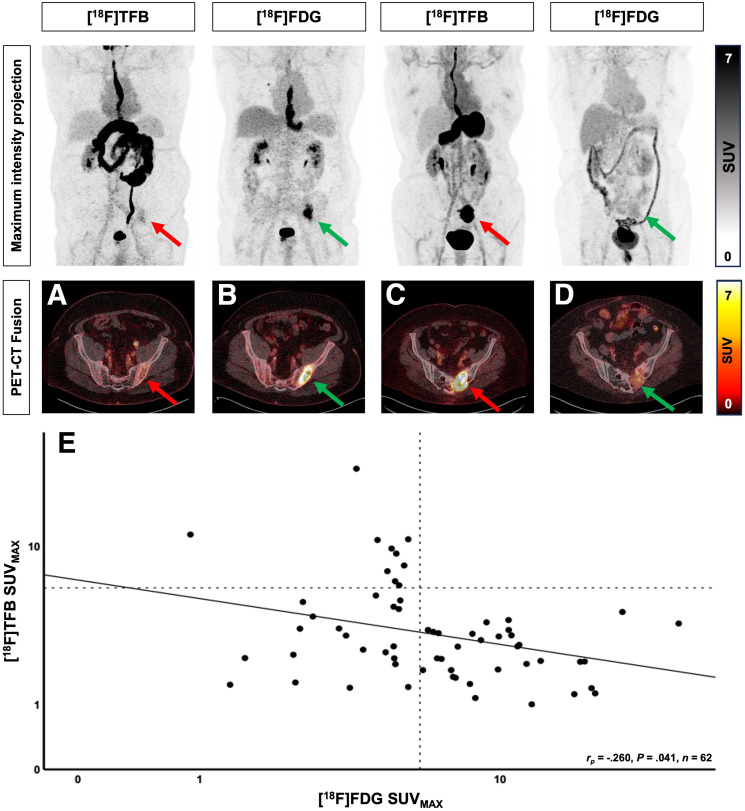
Lesion [^18^F]TFB vs. [^18^F]FDG uptake. 70-y-old man with follicular thyroid carcinoma and singular bone metastasis in left ilium. Dedifferentiation was demonstrated with low [^18^F]TFB uptake (A) and intense [^18^F]FDG uptake (B). 75-y-old woman with follicular thyroid carcinoma and singular differentiated bone metastasis in left-side sacrum. High NIS expression was demonstrated with intense [^18^F]TFB uptake (C) and low [^18^F]FDG uptake (D). (E) Inverse Pearson correlation representing significant inverse relationship between NIS expression (differentiation) and glucose consumption ([^18^F]FDG) in lesion SUV_max_ of [^18^F]TFB and [^18^F]FDG.

The SUV_max_ was measured in all suspected lesions (*n* = 62) to evaluate [^18^F]TFB uptake as a potential surrogate marker of differentiation. The SUV_max_ of [^18^F]TFB was lower and inverse compared with that of [^18^F]FDG (3.97 [95% CI, 3.1–4.9] vs. 6.23 [95% CI, 5.9–9.1]; *P* < 0.001). The SUV_max_ of [^18^F]TFB and [^18^F]FDG showed a statistically significant, intermediate negative correlation (*r*_p_ = −0.26; *P* = 0.041; *n* = 62) (Fig. 4).

### Comparison of [^18^F]TFB PET/CT Acquisition Time Points

Whole-body static imaging was performed 40 and 90 min after [^18^F]TFB administration (*n* = 23; 88%; 49 lesions) to evaluate the tracer kinetics. The SUV_max_ of suspected lesions declined from the 40-min to the 90-min time point (4.18 [95% CI, 3.1–5.3] vs. 3.9 [95% CI, 2.8–4.9]). The Pearson coefficient showed a highly significant, strong linear correlation between both time points (*r*_p_ = 0.94; *P* < 0.001; *n* = 49) (Supplemental Fig. 1). Also, there was a highly significant, strong linear correlation between the 40-min and 90-min time points for normalized lesion uptake: blood-pool TBR (*r*_p_ = 0.88; *P* < 0.001), muscle TBR (*r*_p_ = 0.91; *P* < 0.001), and lung TBR (*r*_p_ = 0.90; *P* < 0.001). Detailed results of quantification of [^18^F]TFB PET at 40 and 90 min after injection are illustrated in Supplemental Table 2.

## DISCUSSION

Despite multiple imaging options, accurate diagnosis of recurrent DTC remains challenging (19). Appropriate therapeutic decisions are especially difficult to make in cases of incipient dedifferentiation or TENIS syndrome (20*,*^21^). Here, 26 patients who received [^18^F]TFB PET/CT and [^131^I]iodine TxWBS-SPECT/CT have been analyzed. The detection rate of both tracers was relatively similar, but slightly more lesions could be identified positive on [^18^F]TFB PET/CT. This indicates that [^18^F]TFB PET/CT might help in the selection of DTC patients for RAI.

[^131^I]iodine diagnostic WBS with SPECT/CT was clearly inferior to [^18^F]TFB PET/CT in our previous study (12). In contrast, [^131^I]iodine TxWBS-SPECT/CT is an excellent imaging modality and has previously not been compared with [^18^F]TFB-based imaging (22). However, patients are exposed to significantly higher radiation levels from [^131^I]iodine, and WBS-SPECT/CT has limited spatial resolution (23). Moreover, RAI has a variety of dose-dependent acute and long-term side effects, like adverse effects on the salivary and lacrimal glands up to secondary malignancies after repeated high activity of RAI (24*,*^25^). Also, it is well known that with increasing [^131^I]iodine activity, more nontumor-related accumulations occur (26*,*^27^). These false-positive findings may lead to unnecessary further invasive procedures as biopsies (Fig. 3). In contrast, lesions with false-positive [^131^I]iodine uptake did not show increased uptake of [^18^F]TFB in our cohort. Considering the detection rates and correct identification of false-positive [^131^I]iodine uptake by [^18^F]TFB, we see great potential in the implementation of [^18^F]TFB PET in the clinical routine before RAI for optimal treatment planning and decision making in recurrent DTC.

Several genetic alterations leading to decreased expression of NIS are the major contributors to RRDTC (28). The decrease in iodine uptake is often correlated with an increase in glucose metabolism, thereby providing a rationale for the higher sensitivity of [^18^F]FDG PET/CT in dedifferentiated disease (16). We show here for the first time that the lesion differentiation can be quantified using [^18^F]TFB and [^18^F]FDG PET/CT with a significant inverse correlation between [^18^F]TFB and [^18^F]FDG uptake. This has previously been demonstrated for [^124^I]iodine and [^18^F]FDG PET/CT (29). However, [^124^I]iodine imaging is only available at a few centers and has many disadvantages in clinical practice (5) like high-energy γ-rays degrading spatial resolution and a long half-life of 4.2 d resulting in higher radiation exposure (30). In contrast, [^18^F]TFB is easy to synthesize in any cyclotron-based radiochemistry and has favorable physical properties for PET imaging (13*,*^31^). The presented data underscores the complexity and heterogeneity of advanced disease in DTC and RRDTC. In particular, TENIS syndrome or dedifferentiation complicates clinical routine and the use of [^18^F]TFB and [^18^F]FDG may establish a new differentiation/dedifferentiation score in DTC.

The biodistribution and kinetics of [^18^F]TFB have already been investigated using dynamic whole-body imaging in healthy adults. The results showed a rapid blood clearance and a good background-to-target ratio of NIS-expressing organs after 30–45 min (10). However, studies illustrating the biodistribution of recurrent DTC metastases are still lacking (32). In the presented study, between 40 and 90 min after injection, maximum TBR values showed a decreasing trend and a significant correlation. This result may simply reflect washout due to nontrapping/nonmetabolization of [^18^F]TFB. Like [^99m^Tc]TcO_4_^−^, [^18^F]TFB is rapidly taken up by the NIS and concentrated in the cell, but it is not metabolized like radioactive iodine (31). Therefore, imaging at a later time point does not appear to be useful or necessary for the detection of DTC lesions.

The present study has some limitations due to its retrospective design and small patient cohort. Due to the heterogeneous disease pattern of recurrent DTC, there were many patients with partially dedifferentiated disease and therefore fewer pure [^18^F]TFB- or [^131^I]iodine-positive findings. Prospective studies in predefined patient cohorts are needed to define the role of [^18^F]TFB PET in recurrent DTC compared with [^131^I]iodine imaging and [^18^F]FDG PET. Change in management due to [^18^F]TFB PET is another point to be addressed needing consistent follow-up and prospective datasets. Thus, to identify potential false-positive enrichments and pitfalls. Response per lesion was not feasible as RRDTC patients undergo different non-RAI therapies in further follow-up.

## CONCLUSION

[^18^F]TFB PET might offer a noninferior diagnostic performance compared with [^131^I]iodine imaging with therapeutic activities. Together with [^18^F]FDG PET, [^18^F]TFB PET might establish a new quantitative measure of differentiation/dedifferentiation for optimal therapeutic management of recurrent DTC with suspicion of dedifferentiation. Further prospective studies on the clinical implementation of [^18^F]TFB are warranted.

## DISCLOSURE

Robert Seifert received research support from Boehringer Ingelheim Fonds and Else Kröner-Fresenius-Stiftung. Philipp Schindler and Wolfgang Roll received funding from the Maria-Möller-Stiftung. No other potential conflict of interest relevant to this article was reported.

## Notes

KEY POINTS **QUESTION:** Does [^18^F]TFB PET/CT provide detection rates in patients with recurrent DTC similar to those provided by the gold standard [^131^I]iodine WBS-SPECT/CT at therapeutic doses? **PERTINENT FINDINGS:** The detection rate of [^18^F]TFB PET/CT in recurrent DTC is comparable to that of [^131^I]iodine WBS-SPECT/CT, with fewer false-positive lesions. [^18^F]FDG PET is recommended as an additional imaging tool to detect dedifferentiation of tumor lesions. **IMPLICATIONS FOR PATIENT CARE:** The pretherapeutic use of [^18^F]TFB PET before radioactive iodine therapy may guide the use of high-dose treatment in patients with recurrent DTC.
